# “Please say what this word is”: Linguistic experience and acoustic context interact in vowel categorization a)

**DOI:** 10.1121/10.0020558

**Published:** 2023-08-09

**Authors:** Christian Stilp, Eleanor Chodroff

**Affiliations:** 1 Department of Psychological and Brain Sciences, University of Louisville, Louisville, Kentucky 40292, USA; 2 Department of Language and Linguistic Science, University of York, York, YO10 5DD, United Kingdom christian.stilp@louisville.edu, eleanor.chodroff@uzh.ch

## Abstract

Ladefoged and Broadbent [(1957). J. Acoust. Soc. Am. **29**(1), 98–104] is a foundational study in speech perception research, demonstrating that acoustic properties of earlier sounds alter perception of subsequent sounds: a context sentence with a lowered first formant (F1) frequency promotes perception of a raised F1 in a target word, and vice versa. The present study replicated the original with U.K. and U.S. listeners. While the direction of the perceptual shift was consistent with the original study, neither sample replicated the large effect sizes. This invites consideration of how linguistic experience relates to the magnitudes of these context effects.

## 1. Introduction

The phonetic realization of vowels can vary considerably from talker to talker, from dialect to dialect, and even from generation to generation [e.g., Peterson and Barney (1952), Hillenbrand *et al.* (1995), Clopper *et al.* (2005), Ferragne and Pellegrino (2010), and Fox and Jacewicz (2019)]. Despite this extreme acoustic variability, most listeners are highly proficient in understanding speech, even for novel talkers or in adverse listening conditions. Proficiency in identifying a given speech sound stems in large part from a listener's ability to use intrinsic acoustic properties of the speech sound as well as extrinsic acoustic properties of surrounding speech sounds [e.g., Ainsworth (1975), Nearey (1989), and Stilp (2020)]. A classic example of speech perception in context was provided by Ladefoged and Broadbent (1957). Listeners heard different renditions of the sentence “Please say what this word is” with F1 or F2 values shifted up to higher frequencies or down to lower frequencies. On each trial, the context sentence was followed by a target word that listeners identified as “bit,” “bet,” “bat,” or “but.” Notably, for an ambiguous “bit”–“bet” target word, when F1 frequencies in the context sentence were shifted upwards, listeners were more likely to hear the target word as low-F1 “bit”; when F1 frequencies in the sentence were shifted downwards, listeners were more likely to hear high-F1 “bet.” Thus, speech perception does not operate solely on acoustic properties of each individual sound, but also considers how those properties relate to the acoustic properties of the surrounding context.

The findings from Ladefoged and Broadbent (1957) generated a tremendous amount of research into how speech perception is modulated by acoustic context. However, studies exploring acoustic context effects on the perception of the /ɪ/-/ɛ/ contrast generally reported much smaller effect magnitudes than the extremely large shifts in Ladefoged and Broadbent (1957). In that study, U.K. English listeners labeled a canonical [bɛt] target word as “bit” when preceded by a high-F1 context sentence 97% of the time, and as “bet” when preceded by a neutral-F1 context sentence 92% of the time. Ladefoged's (1989) replication study using natural speech on U.S. listeners in California found a slight decrease in the magnitude, but one that was still relatively large: 75% of listeners heard an ambiguous [bɪt]–[bɛt] target word as “bit” when preceded by a high-F1 context sentence and as “bet” when following a low-F1 context sentence. A host of experiments with U.K. English listeners reported by Watkins (1991) and Watkins and Makin (1994) saw perceptual shifts from /ɪ/ to /ɛ/ that ranged from very small to extremely large and depended considerably on the exact acoustic context (e.g., presence or absence of a silent gap between context and target, monotic or dichotic presentation, speech or signal-correlated noise context, forward or backward presentation of the context). Similarly, experiments with Dutch and U.S. English listeners also found that characteristics of the acoustic context modulated the magnitude of the perceptual shift from /ɪ/ to /ɛ/; however, the perceptual shifts only ever reached an upper limit of approximately 35% to 40% for a given vowel continuum member [e.g., presence or absence of pitch flattening, time reversals, spectral rotation, among others for Sjerps *et al.* (2011) and Sjerps *et al.* (2013); e.g., gain and bandwidths of filters amplifying the F1 region for Assgari and Stilp (2015) and Stilp *et al*. (2015)]. Finally, Bourguignon *et al.* (2016) tested Canadian English listeners on an /ɪ/–/ɛ/ continuum anchored by the original [bɛt] target word and preceded by the original context sentences from the study of Ladefoged and Broadbent (1957). The difference in the perception of “bet” between the high- and low-F1 context sentences was only 19% for the original [bɛt] target word (cf., 93% for the participants in the original study). Numerous factors could have contributed to this range of findings, including but not limited to variability in stimuli, listeners, dialects, generations, or testing methods.

The primary objective of this study was to replicate Ladefoged and Broadbent (1957) as closely as possible to better understand factors underlying this discrepancy in perceptual shift magnitudes. We obtained and tested digitized versions of the original stimuli from the 1957 study. Two native English adult listener samples were tested: one in the United Kingdom and another in the United States, to test for potential influences of regional native language background on perception. Comparing patterns of results from the original study (conducted in 1956) to new data (collected in 2022) also highlights potential generational differences in speech perception.

In the analysis presented here, we focus on the perception of the original [bɛt] target word, henceforth the neutral-F1 target word, following the high-, neutral-, and low-F1 context sentences, which is the most referenced aspect of the study of Ladefoged and Broadbent (1957). We hypothesize that categorization of the target word will be significantly modulated by the F1 of the context sentence. Specifically, the high-F1 context sentence will result in an increased “bit” response, and a low-F1 context sentence, an increased “bet” response. Based on previous findings, we expect all participant groups to demonstrate the same directional shift, but for U.K. listeners to exhibit larger perceptual shifts than U.S. listeners due to the accordance between their native language background and that of the talker who produced the stimuli.

## 2. Methods

### 2.1 Participants

**U.K. 1957:** 60 participants were tested in the U.K. Of these, 19 participants had an English Received Pronunciation (RP) accent, 19 an English-influenced Scots accent, and 7 a Scots accent; the accents of 15 participants were unaccounted for in the original article. Detail relating to gender or regional background was also not reported. **U.K. 2022**: 33 native speakers of British English participated at the University of York in York, U.K. in Spring 2022 (27 female, 6 male; 19 to 27 years old, median 20 years old). Data from an additional 14 participants were collected but excluded on the basis of having grown up outside the U.K. or being a non-native speaker (n = 12), or reporting impaired hearing (n = 2). Of the 33 participants, 12 were from Northern England (6 East, 6 West), 11 were from the Midlands (11 East, 1 West), 7 were from Southern England (5 East, 2 West), and 1 was from Scotland and Northern England. **U.S. 2022**: 28 native speakers of American English participated at the University of Louisville in Kentucky, USA in Spring 2022 (19 female, 8 male, 1 unreported; 18 to 30 years old, median 20 years old). Of these, 20 were from Kentucky, 5 from neighboring states (2 Illinois, 1 Indiana, 1 Ohio, 1 Tennessee), 1 from Florida, 1 from Utah, and 1 from North Dakota. Data from one additional participant was excluded as English was not the reported L1. Additional participant information for the 2022 groups is included in an online repository (Stilp and Chodroff, 2023).

### 2.2 Stimuli

Digitized versions of the original Ladefoged and Broadbent (1957) stimuli were provided by the University of Edinburgh Linguistics and English Language Department, who had undertaken the effort to digitize “moldering” audio tapes in the lab (Ladd *et al.*, 2021); all stimuli are available at Stilp and Chodroff (2023). All stimuli were spoken in RP by a male speaker from Southern England in his early 30s (Peter Ladefoged) and were recorded around the year 1956. The stimuli comprised six context sentences and four target words from the original Ladefoged and Broadbent (1957) study. The context sentences were resynthesized from the original recording using the Parametric Artificial Talking (PAT) Device (Lawrence, 1955) and modulated along two primary dimensions of F1 and F2 with an additional simultaneous F1+F2 modification. This resulted in the following context sentences: neutral F1+neutral F2, low F1, high F1, low F2, high F2, and low F1+high F2. The target words were meant to fall in the region of the vowel space occupied by the vowels “bit,” “bet,” “bat,” or “but.” The primary manipulations were also along the F1 and F2 dimensions, resulting in the following target words: low F1, neutral F1+high F2, high F1, and low F2. These four words, respectively, corresponded to Ladefoged and Broadbent's target words A, B, C, and D. F1 and F2 values of the acoustic context and target words can be found in the supplementary material of the present study^1^ and in the original Ladefoged and Broadbent study.

The pairings of context sentences and target words targeted modifications along the F1 dimension, the F2 dimension, and finally a simultaneous F1+F2 change; however, the context sentences and targets were not fully crossed. A total of 11 pairings were presented and consisted of seven F1 target and context manipulations (of which only the three neutral-F1 target word trials are assessed), four F2 target and context manipulations (one of which overlapped with the F1 manipulations), and two simultaneous F1+F2 manipulations (one of which overlapped with the F2 manipulations). The present analysis focuses on the perception of the neutral-F1 target word (F1 = 450 Hz) following the high-F1 (F1 = 380–660 Hz), neutral-F1 (F1 = 275–500 Hz), and low-F1 (F1 = 200–380 Hz) context sentences.

### 2.3 Procedure

The 11 stimuli were presented in the same order to all local participants with one additional practice trial at the beginning. For each trial, the response options were “bit,” “bet,” “bat,” or “but.” The practice trial was a neutral F1+neutral F2 context sentence paired with the low-F1 target word. **U.K. 1957**: Participants marked their responses on answer sheets which the experimenters provided. The experiment was presumably held in a lecture theater with stimuli presented over loudspeakers, which we infer was the methodology given its use in a later study by this group (Broadbent and Ladefoged, 1960). **U.K. 2022**: Participants completed the experiment in the context of a phonetics and phonology class held in a medium-sized tiered lecture theater with stimuli presented over loudspeakers (capacity = 198 people). Responses were entered into a Google form. **U.S. 2022**: 19 participants completed the experiment within the context of a statistics class held in a medium-sized tiered lecture theater with stimuli presented over loudspeakers (capacity = 148 people). The remaining 9 participants completed the experiment within the context of a psychology laboratory meeting in a small classroom environment with stimuli presented over the classroom loudspeakers (capacity = 20 people). For both U.S. samples, responses were entered into a Google form. Responses did not statistically differ between these two groups, so these were pooled into a single sample.

### 2.4 Statistical analysis

For this analysis, we concentrated on the balanced component of the original experiment involving the neutral-F1 target word preceded by the low-, neutral-, and high-F1 context sentences. Full analyses of the remaining trials (F1, F2, and simultaneous F1+F2 manipulations) can be found in the supplementary material^1^ and Stilp and Chodroff (2023).

A fixed-effects binary logistic regression was implemented to predict the categorization of “bit” (coded as 1) or “bet” (coded as 0) as a function of the F1 context sentence (low, neutral, or high), participant group (U.K. 1957, U.K. 2022, or U.S. 2022), and their interactions. A binary logistic regression was chosen over a multinomial or ordinal regression as 99% of responses to the stimuli of interest were either “bit” or “bet” (3 of 362 responses were “bat”). Random effects were not included for participant or item as neither stimuli nor conditions (i.e., context–target pairing) were ever repeated. Context sentence was treatment coded with neutral serving as the baseline level; the model was rerun with low serving as the baseline level to contrast results across low-F1 and high-F1 context conditions. Participant group was also treatment coded with each level serving as a baseline in separate models to allow for full statistical comparison of the relevant hypotheses. Unless otherwise stated, the model coefficients can be interpreted relative to the U.K. 1957 group in the neutral-F1 context condition.

## 3. Results

**Fig. 1.**
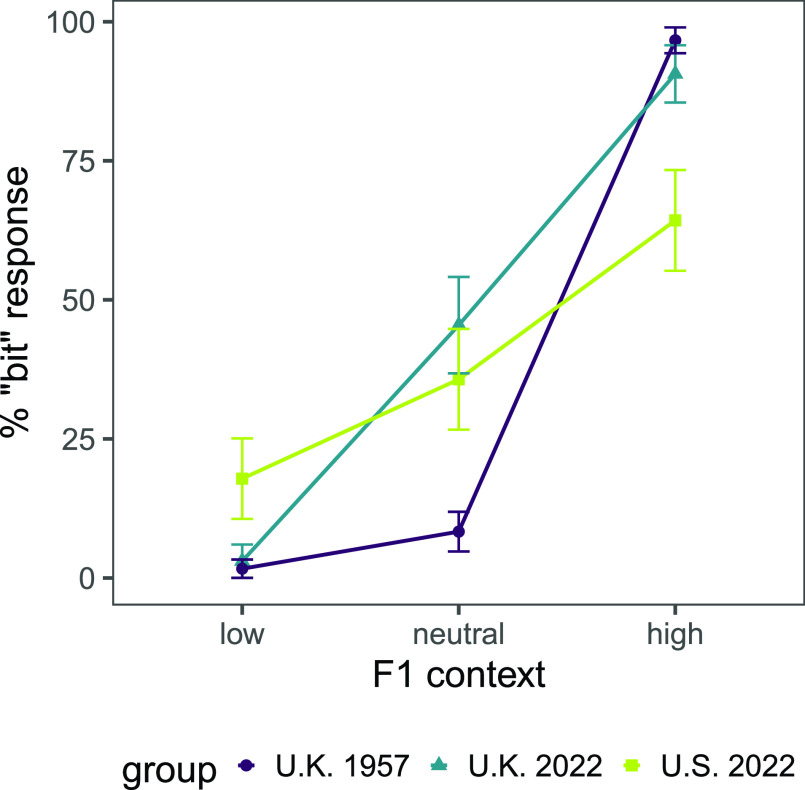
Percent “bit” response across the three F1-modulated context sentences from each group (U.K. 1957, U.K. 2022, and U.S. 2022).

Overall, significant differences were observed across all three groups in response to the neutral-F1 target word following all three contexts. Compared to the U.K. 1957 group, the two 2022 groups had significantly smaller shifts from “bet” to “bit” from the neutral to high context, and the U.S. 2022 group had more tempered shifts from “bet” to “bit” across the low and high contexts (18% to 64% “bit” for U.S. 2022 vs 2% to 97% “bit” for U.K. 1957 and 3% to 91% “bit” for U.K. 2022; see Fig. 1).

More specifically, in the neutral-F1 context condition, the U.K. 1957 group was significantly less likely to respond “bit” than “bet,” whereas the U.K. 2022 group and the U.S. 2022 group responded “bit” and “bet” more equally (U.K. 1957 intercept: *β* = −2.40, *p* < 0.001; U.K. 2022 intercept: *β* = −0.18, *p* = 0.60; U.S. 2022 intercept: *β* = −0.59, *p* = 0.14). Moreover, relative to the U.K. 1957 group, both 2022 groups responded “bit” significantly more in the neutral condition (U.K. 2022: *β* = 2.22, *p* < 0.001; U.S. 2022: *β* = 1.81, *p* < 0.01). The 2022 groups did not differ significantly from each other (U.K. 2022 vs U.S. 2022: *β* = −0.41, *p* = 0.44).

In the low-F1 context condition, the “bit” response rate decreased significantly for the U.K. 2022 group relative to the neutral context (U.K. 2022 low context: *β* = −3.28, *p* < 0.01). The U.K. 1957 and U.S. 2022 groups responded “bit” numerically but not significantly less often following the low context than the neutral context (U.K. 1957 low context: *β* = −1.65, *p* = 0.14; U.S. 2022 low context: *β* = −0.94, *p* = 0.14). Though the response patterns between the two 2022 groups trended towards significance in the change from neutral to low contexts (U.K. 2022 vs U.S. 2022 low context: *β* = 2.35, *p* = 0.06), none of the groups differed significantly from one another (U.K. 1957 vs U.K. 2022 low context: *β* = −1.64, *p* = 0.29; U.K. 1957 vs U.S. 2022 low context: *β* = 0.71, *p* = 0.58).

In the high-F1 context condition, each group responded “bit” more often following the high context relative to the neutral context (U.K. 1957 high context: *β* = 5.77, *p* < 0.001; U.K. 2022 high context: *β* = 2.45, *p* < 0.001; U.S. 2022 high context: *β* = 1.28, *p* < 0.05). However, the magnitude of the effect differed significantly between groups. Relative to the large increase in “bit” responses across neutral and high contexts for the U.K. 1957 group, both 2022 groups had a significantly smaller increase in “bit” responses (U.K. 1957 vs U.K. 2022 and high context: *β* = −3.31, *p* < 0.01; U.K. 1957 vs U.S. 2022 and high context: *β* = −4.48, *p* < 0.001). While the 2022 groups' shifts did not significantly differ from one another relative to the neutral context (U.K. 2022 vs U.S. 2022 and high context: *β* = −1.17, *p* = 0.19), the U.S. 2022 group had a significantly smaller increase in “bit” responses from the low to the high context relative to the U.K. 2022 group (U.K. 2022 vs U.S. 2022 and high vs low context: *β* = −3.52, *p* < 0.01). In addition, the U.S. 2022 group differed significantly from the U.K. 1957 group in the change from low to high context (U.K. 1957 vs U.S. 2022 and high vs low context: *β* = −5.19, *p* < 0.001), indicating a significantly more tempered change relative to the two U.K. groups.

Finally, significant differences were also observed among the U.K. 1957, U.K. 2022, and U.S. 2022 groups for the remaining trials involving additional F1, F2, and simultaneous F1+F2 manipulations (see supplementary material^1^).

## 4. Discussion

The primary motivation for the present study was to explore the magnitudes of perceptual shifts reported in Ladefoged and Broadbent (1957). While some methodological details were absent from the original study, we pursued a good-faith replication using digitized versions of the original stimuli and likely methods. Focusing on the context effects involving the neutral-F1 target word, shifts were dramatic for the original listeners (data labeled U.K. 1957), not nearly as dramatic for contemporary U.K. listeners (U.K. 2022), and comparatively small for U.S. listeners (U.S. 2022).

Best practices in experimental methodology have evolved since the seminal report by Ladefoged and Broadbent (1957). Various methodological details and formal statistical analyses were missing in the original report [and from contemporary reports from that research group; Broadbent *et al.* (1956) and Broadbent and Ladefoged (1960)]. Moreover, the original study utilized a “one-shot” design where each token was tested once; it is now common practice to test multiple replications of each token to estimate any potential variability per individual. Finally, while details of the testing environment were omitted from the original study, we inferred that listeners were tested simultaneously in larger rooms. Many investigators nowadays might elect to test listeners individually over headphones in sound-treated spaces to limit experimental noise. Even in our efforts to replicate Ladefoged and Broadbent (1957) as closely as possible, perceptual shift magnitudes still varied dramatically across listener groups, potentially pointing to non-methodological factors that underlie these differences in performance.

Considerable research has shown that short-term acoustic contexts significantly influence subsequent speech perception, and these contrast effects may be rooted in general auditory mechanisms of perception [see Stilp (2020) for review]. The present study is indeed compatible with a role of spectral contrast: all participant groups demonstrated the same direction of shifts as would be predicted by this general auditory influence. However, spectral contrast alone cannot account for the fact that the participant groups demonstrated shifts of differing magnitudes. Additional top-down influences likely contribute to these results as well; however, studies on the interaction between linguistic experience and spectral contrast effects have produced mixed results. Sjerps and Smiljanić (2013) reported comparable shifts from context sentences (in Spanish, English, or Dutch) on categorization of /o/ and /u/ by English, Dutch, Spanish, and Spanish-English bilingual listeners. Conversely, Kang *et al.* (2016) reported differential effects of context for French and English listeners' categorization of /s/ and /ʃ/ when followed by the vowels /a/, /u/, or the French vowel /y/. French listeners have extensive experience with all three and thus responded differentially to each, but English listeners' significantly less experience with French /y/ elicited similar amounts of /s/ responses to [sy] as [sa]. One direction for future studies could be to examine the precise relationship between the participant's production and perception of the implicated categories. It bears mention that language background is not the only top-down influence on spectral contrast effects; both selective attention (Bosker *et al.*, 2020) and expectations of talker characteristics (Johnson *et al.*, 1999) have been reported to influence the magnitudes of these effects.

The context–target trial structure has been used to explore higher-level influences on perception as well. Several studies have examined how the accent of context sentences shaped perception of the target sound/word. Evans and Iverson (2004) measured goodness ratings of synthesized vowels embedded in context sentences spoken in Sheffield or Standard Southern British English accents. The best-rated exemplars of [bʊd] and [kʊd] had different F1 frequencies depending on the accent of the context sentence (lower F1 in Sheffield context sentences), with larger accent effects for listeners from northern England. When repeating this task with northern English listeners before and during their attendance at a university in Southern England (London), their vowel production progressively changed to match university norms but their goodness ratings for synthesized vowels in context sentences did not change over time (Evans and Iverson, 2007). Listeners in Floccia *et al.* (2006) completed a lexical decision task for disyllabic targets at the end of context sentences spoken in different French regional accents. Context sentences spoken in an unfamiliar accent elicited delays in lexical decisions compared to those spoken in familiar accents. However, across these studies, acoustic properties of the context sentence and how they related to acoustic characteristics of the target items were not reported. While these studies focused on top-down effects of regional accent, one cannot rule out potential contributions from lower-level acoustic context of surrounding sounds as well. Future research in multiple disciplines would be strengthened by reporting full acoustic details of context and target stimuli in order to account for each of these influences separately.

The present results reflect a combination of lower-level, bottom-up contributions to speech perception (signal acoustics, spectral contrast effects) with higher-level, top-down contributions (dialect, generation). Spectral contrast effects were observed for all three listener groups, but exactly when those shifts occurred and their relative magnitudes were heavily influenced by the relationship between signal acoustics and experiential factors such as dialect. This interplay is offered as an at least partial explanation why perceptual shifts in Ladefoged and Broadbent (1957) have proven well-replicated in kind but not in magnitude. Thus, it is essential to consider not only how acoustic properties of context stimuli relate to acoustic properties of target stimuli, but also how these acoustics relate to the listeners and their linguistic backgrounds. Moving forward, researchers should be mindful to document relevant acoustic properties of the stimuli, the linguistic backgrounds of the participants, and participate more generally in open science to facilitate subsequent replications and extensions.

## Footnotes

^1^
See supplementary material at https://doi.org/10.1121/10.0020558 for full analyses of all experimental conditions and trials.
